# Septic pulmonary embolism caused by postpartum ovarian vein thrombophlebitis: A case report

**DOI:** 10.1016/j.crwh.2022.e00445

**Published:** 2022-08-19

**Authors:** Mintita Pumtako, Chattarin Pumtako

**Affiliations:** a Somdej Phrachao Taksin Maharat Hospital, 16/2 Phahonyothin Road, Rahaeng Subdistrict, Mueang Tak District, Tak 63000, Thailand; b University of Glasgow, G12 8QQ Glasgow, United Kingdom; c Chulabhorn Royal Academy, 906 Thung Song Hong, Lak Si, Bangkok, 10210, Thailand

**Keywords:** Ovarian vein thrombophlebitis, Postpartum fever, Septic pulmonary embolism, Case report

## Abstract

**Objective:**

To report on the clinical considerations for and management of ovarian vein thrombophlebitis (OVT). OVT is an element of septic pelvic thrombophlebitis (SPT). OVT is a relatively uncommon cause of postpartum fever. It manifests in approximately 0.01–0.02% of vaginal deliveries and 0.1% of caesarean births. A delay in diagnosis and treatment can lead to potentially fatal complications.

**Case Presentation:**

A 38-year-old woman presented to the hospital with a fever and dyspnoea. She had had a spontaneous home birth and developed a low-grade fever after delivery. The infant died 1 h after birth due to an unknown cause. After 72 h of intravenous antibiotic treatment, the patient's condition did not improve. Postpartum endometritis with pneumonia was suspected. OVT was also suspected. The patient received broad-spectrum antibiotics and anticoagulant therapy. After 7 days of treatment, repeat computed tomography scan revealed that the condition of the lungs and uterus had improved.

**Conclusion:**

Puerperal fever and septic pulmonary embolism may be signs of OVT. Thus, a diagnosis of OVT should be considered when a patient presents with prolonged fever postpartum and is not responsive to standard endometritis therapy. Moreover, anticoagulant therapy can confirm the diagnosis if the fever subsides following broad-spectrum antibiotic treatment.

## Highlights

• Ovarian vein thrombophlebitis (OVT) can cause complications such as septic pulmonary embolism and lung abscesses.
• OVT should be considered when a patient with prolonged fever postpartum not responsive to standard endometritis therapy.
• If the fever subsides following broad-spectrum antibiotic and anticoagulant treatment, the diagnosis of OVT can be confirmed.
• It is important to conduct more studies on the effectiveness and duration of anticoagulant therapy for OVT.

## 1 Introduction

Septic pelvic thrombophlebitis (SPT) is a well-known but infrequent postpartum complication (occurring in about 1 in 3000 deliveries). It is more prevalent after caesarean delivery (1/800 versus 1/9000 cases after vaginal delivery), most likely because caesarean sections increase the risk of related puerperal infections [1]. SPT was previously quite common; its treatment was almost entirely surgical, and it was associated with a high mortality rate. Although the prognosis for SPT has recently improved, it can still result in life-threatening complications [2].

Ovarian vein thrombophlebitis (OVT) is an element of SPT that is a relatively uncommon cause of postpartum fever. It presents in approximately 0.01–0.02% of vaginal deliveries and 0.1% of caesarean births [3]. When a condition is uncommon, diagnosis and proper treatment may be delayed, leading to potentially fatal complications such as sepsis, inferior vena cava thrombosis and septic pulmonary embolism [4].

This case report describes the diagnosis and management of postpartum septic pulmonary embolism caused by ovarian thrombophlebitis in a low-resource setting.

## 2 Case presentation

A 38-year-old woman presented to the hospital with a fever and dyspnoea. Her history was obtained from her husband. He did not know about her underlying diseases, and she had no history of drug allergies or surgical treatment and no family history of thrombotic diseases. Not having received antenatal care, she had had a spontaneous home birth and developed a low-grade fever after delivery. The infant died 1 h after birth due to an unknown cause. On the second day following the vaginal delivery, the patient developed a foul odour from the vagina and bloody vaginal discharge but did not seek treatment. On the fifth day after delivery, the patient developed a high-grade fever. The antipyretic acetaminophen was administered but her condition did not improve; her husband then brought her to the hospital that same day. Her most recent period had been eight months prior. She had two sons, aged 5 and 2 years, who were both born at home and were healthy.

At the emergency room, her temperature was 38.6 °C, pulse rate 121 bpm, blood pressure 101/58 mmHg and room-air oxygen saturation 94%. On examination, the lungs, abdomen and legs were all normal. The patient experienced suprapubic pain without rebound tenderness. Blood analysis revealed a white blood cell count of 20,740/L with a neutrophil percentage of 89.4%. A urine test was normal. An ultrasound scan of the pelvis was negative. The presumptive diagnosis was endometritis. Intravenous ampicillin and gentamycin were administered before the patient was admitted to the obstetrics and gynaecology ward. Signs of vaginal delivery were detected by per vagina examination, with foul-smelling lochia and swollen perineum observed by the obstetrician. After 72 h of intravenous antibiotic treatment, the patient's condition did not improve. However, the room-air oxygen saturation was 96% despite her continuing fever and dyspnoea.

A physician specialising in infectious diseases was consulted, and additional investigations were considered. Blood analysis revealed a negligible change in the white blood cell count, which was now at 15,730/L with 85.2% neutrophils. Three days of negative haematology were followed by a positive sputum culture for moderate *Klebsiella pneumoniae*. Postpartum endometritis with pneumonia was suspected. Endocarditis was evaluated with an echocardiogram, and the result was normal. The antibiotic therapy was changed to 2 g of meropenem every 8 h. Despite two days of meropenem treatment, the patient's fever continued to increase. A CT scan was requested to investigate additional causes of prolonged fever.

**Fig. 1:**
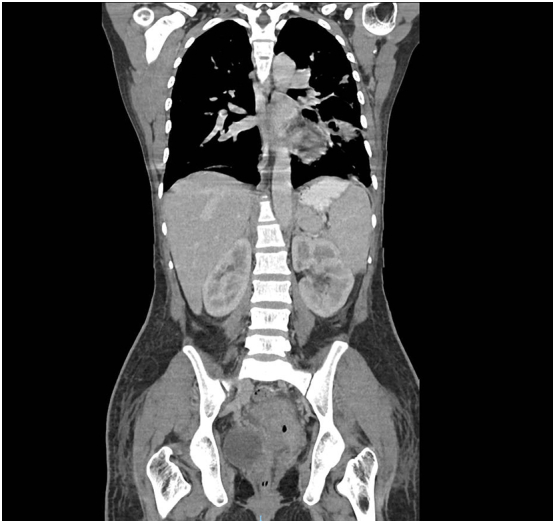
A CT scan demonstrating central cavitation in the superior segment of the left lower lung suggests a septic pulmonary embolism.

The CT scan revealed multiple small nodular opacities with associated ground-glass opacities scattered bilaterally in the lungs. Some of these lesions showed central cavitations along with some abscess formation and bilateral pleural effusion. Septic pulmonary embolism was suspected (Fig. 1). The enlarged size of both ovaries and the uterus with heterogeneous enhancement, minimal free fluid and few air bubbles in the uterine cavity, as well as multiple small rim-enhancing hypodense lesions at the right-side uterine wall, suggested endometritis with abscesses. Moreover, the uterus was suspected to be enlarged postpartum, and the CT scan confirmed this suspicion.

**Fig. 2:**
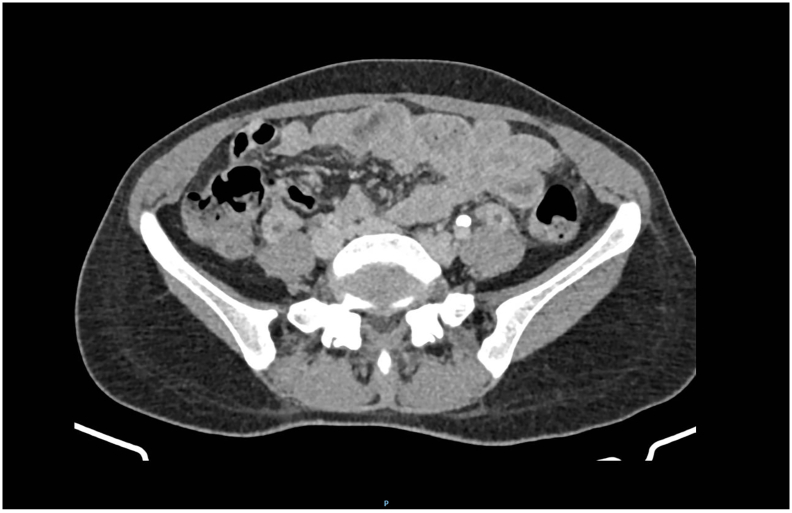
The ovarian vein is thick-walled and enlarged, with central tubular hypodensity and rim enhancement, which is indicative of ovarian vein thrombosis.

Based on these findings, SPT with complications became a concern. The patient received broad-spectrum antibiotics (2 g of meropenem every 8 h) and 0.1 mg/kg of enoxaparin twice daily. After 2 days of combined antibiotic and anticoagulant treatment, a positive clinical response was observed, with no worsening of her fever or dyspnoea. The patient's room-air oxygen saturation was 97–99%. After 7 days of anticoagulant therapy, a repeat CT scan revealed that the condition of the lungs and uterus had improved (Fig. 2). The infectious process of the lungs and uterus and the size of the pulmonary septic embolism had decreased, and pleural effusion was absent. The uterus was mildly enlarged with homogeneous enhancement, and a small thrombus was observed in both ovarian veins. As a result, the patient's blood quality had also improved (white blood cell count of 7850/L with 78.5% neutrophils). The patient was discharged in a stable condition with a prescription for 1 g of amoxicillin-clavulanate twice daily for 7 days and 3 mg per day of oral warfarin (target international normalized ratio 2–3) for 6 weeks. At the 6-week follow-up appointment, the patient was doing well.

## 3 Discussion

OVT is a clinical manifestation of SPT. OVT is associated with Virchow's triad, which consists of a hypercoagulable state, stagnant blood flow and injury to the pelvic vein due to spreading uterine infection, which can occur in the postpartum period as a result of birth trauma (5; 6).

The classic triad of OVT symptoms includes fever, lower abdominal pain and a rope-like or sausage-like abdominal mass [4]. If the infected thrombus breaks off, OVT can cause life-threatening complications such as septic pulmonary embolism and lung abscesses [7]. Due to advances in diagnostic technology and earlier diagnosis, the incidence of septic pulmonary embolism has decreased from 13% to 2.7% [4].

For the diagnosis of OVT, imaging modalities such as ultrasonography, CT and magnetic resonance imaging (MRI) must be used. With ultrasonography, a tubular, strip-shaped, hypoechoic lesion is visible adjacent to the abdominal aorta or inferior vena cava when OVT is present, extending upward from the lateral side of the left or right ovary. However, ultrasonography has limited value because the ovarian vein is located in the retroperitoneum and intestinal gas can obscure it [8]. A thick-walled, enlarged ovarian vein with a filling defect will be visible on a CT scan [9]. The thrombus vessel will appear bright on an MRI scan, whereas normal blood flow will appear dark [5]. Dextrorotation of the puerperal enlarged uterus compresses the right ovarian vein and causes retrograde flow from the left to the right system. The right side of the ovarian vein is involved in up to 90% of OVT cases [6].

In the past, OVT was treated with surgical ovarian vein ligation, which also served to prevent pulmonary embolism. The mortality rate decreases from 50% to 4.4% following ligation of the inferior vena cava and ovarian vein [4]. Currently, most cases of OVT are treated with drugs. The current Canadian guidelines advise the continuation of parenteral broad-spectrum antibiotics for a minimum of 48 h before defervescence and clinical improvement. In addition, therapeutic-dose anticoagulation may be considered for 1 to 3 months [10]. However, these recommendations are based solely on clinical experience and expert opinion; the effect of anticoagulants on patients with OVT is the subject of only one small randomised controlled trial, in which one group received anticoagulants and the other did not. There were no differences between the groups regarding the time required to become afebrile or the incidence of thrombotic events [5]. Currently, there is no standard protocol for treating OVT. More studies should be conducted on the effectiveness and duration of anticoagulant therapy for OVT.

In conclusion, a diagnosis of OVT should be considered when a patient presents with prolonged fever postpartum and is not responsive to standard endometritis therapy. Moreover, anticoagulant therapy may confirm the diagnosis of OVT if the fever subsides following broad-spectrum antibiotic and anticoagulant treatment.
